# Elimination of *Rhodnius prolixus *in Central America

**DOI:** 10.1186/1756-3305-5-45

**Published:** 2012-02-22

**Authors:** Ken Hashimoto, Christopher J Schofield

**Affiliations:** 1 1-6-3 Miyakodai, Kamiso, Kakogawa, Hyogo 675-1215, Japan; 2 Department of Infectious and Tropical Diseases, London School of Hygiene and Tropical Medicine, London WC1 E7HT, UK

**Keywords:** Chagas disease, American trypanosomiasis, *Rhodnius prolixus*, vector control, indoor residual spraying, elimination, Central America

## Abstract

*Rhodnius prolixus *is one of the main vectors of *Trypanosoma cruzi*, causative agent of Chagas disease. In Central America, it was first discovered in 1915 in El Salvador, from where it spread northwest to Guatemala and Mexico, and southeast to Nicaragua and Costa Rica, arriving also in Honduras in the late 1950s. Indoor residual spraying (IRS) by the antimalaria services of Costa Rica prevented its spread southwards, and similar IRS programmes appear to have eliminated it from El Salvador by the late 1970s. In 1997, by resolution of the Ministers of Health of the seven Central American countries, a multinational initiative against Chagas disease (IPCA) was launched with one of the specific objectives being the elimination of *R. prolixus *from the region. As a result, more and more infested areas were encountered, and progressively sprayed using an IRS strategy already deployed against *Triatoma infestans *in the southern cone countries of South America. In 2008, Guatemala became the first of these countries to be formally certified as free of Chagas disease transmission due to *R. prolixus*. The other infested countries have since been similarly certified, and none of these has reported the presence of *R. prolixus *since June 2010. Further surveillance is required, but current evidence suggests that *R. prolixus *may now been eliminated from throughout the mesoamerican region, with a corresponding decline in the incidence of *T. cruzi *infections.

## Introduction

By August 2011, all the previously endemic countries of Central America had been formally certified as free of Chagas disease transmission due to their main domestic vector, *Rhodnius prolixus*. None of these countries, nor Mexico, has reported the presence of this vector since June 2010, suggesting that *R. prolixus *may now have been completely eliminated from the mesoamerican region. This is not to say that Chagas disease itself has been eliminated, since there is not only a residue of previously infected cases, but there is also active transmission in some areas due to other vector species - especially *Triatoma dimidiata*. Nevertheless, the results suggest that the burden of transmission has been substantially reduced. This review summarises the background and progress of the multinational initiative against Chagas disease transmission carried out in the Central American countries (known as IPCA - Iniciativa de los Países de Centro América para la Interrupción de la Transmisión Vectorial, Transfusional y Atención Médica de la Enfermedad de Chagas).

**Figure 1:**
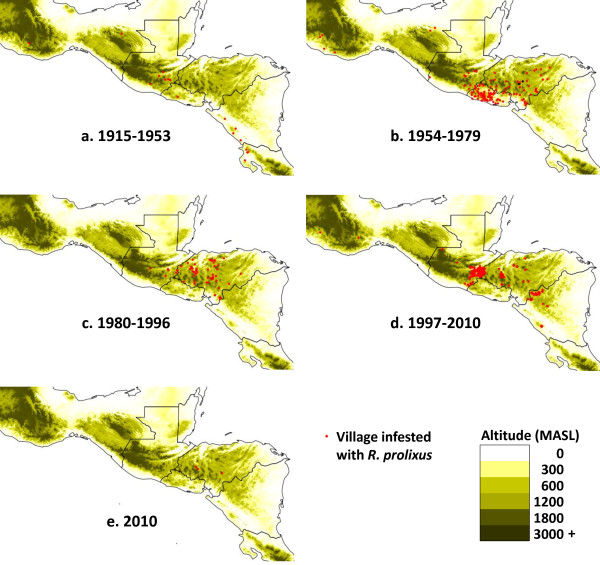
**Changes in the distribution of *Rhodnius prolixus *in Mesoamerica**. a - 1915-1953, first encounter in El Salvador and initial spread NW into Guatemala and Mexico, and SE into Nicaragua and Costa Rica; b - 1954-1979, further spread into Honduras, but elimination from Costa Rica; c - 1980-1996, limited reports due to political unrest; d - 1997-2010, extended surveys following launch of IPCA initiative, accompanied by IRS campaigns in Guatemala, Honduras, El Salvador and Nicaragua; e - 2010, the last four foci remaining in Honduras. Since June 2010, there have been no further reports of *R. prolixus *in Mesoamerica.

Information for the review has come from published scientific articles, reports of national Chagas disease control programmes, reports and presentations of annual meetings of the IPCA initiative, and personal communications. In mapping the historical distribution of *R. prolixus *(Figure 1) the location of some areas or villages was only approximate - some recent documents offer exact coordinates of infested villages, but most publications prior to the mid-1990s identified localities only by administrative departments or municipalities and for these, geographically centric points were selected.

## Background

*Rhodnius prolixus *(Hemiptera, Reduviidae, Triatominae) is one of the most efficient vectors of *Trypanosoma cruzi*, the causative agent of Chagas disease. It is assumed to have evolved from the ancestral forms of other Rhodniini in or around the Amazon region of South America, becoming highly adapted to domestic and peridomestic habitats - especially in the llanos of Venezuela and Colombia, where it remains a significant domestic vector of *T. cruzi *[1]. Historically however, its distribution has shown a notable discontinuity since, although widespread in parts of Venezuela and Colombia, it has never been reported from Panama nor southern or central Costa Rica [2,3].

In Central America, *R. prolixus *was first reported in 1915 from the city of San Salvador [4], from where it subsequently spread in El Salvador and into Guatemala, Honduras, Nicaragua, Costa Rica, and southern Mexico. It is believed that the original specimens in San Salvador resulted from a "laboratory accident". *R. prolixus *had been collected from the region of La Guayra in Venezuela in 1912 and taken to Paris, France, for studies of its potential use in xenodiagnosis of Chagas disease (the feeding of laboratory-reared uninfected bugs on patients, and subsequent examination of the bugs' rectal contents for evidence of parasite multiplication). A sample from the Paris colony was then taken to San Salvador for further studies of its use in xenodiagnosis, and is assumed to have been accidentally released in 1913 [5]. In genetic terms, this scenario implies a series of founder effects and genetic bottlenecks, that would have led to the Central American form of *R. prolixus *being genetically impoverished, as indicated by its relatively small body size and reduced RAPD banding profiles [6]. One consequence is that the Central American form of *R. prolixus *seems to have been unable to colonise silvatic habitats, and appears to have remained in domestic and peridomestic habitats throughout its spread in Central America and Mexico. Nevertheless, it was able to build up very large domestic populations, with over 11,000 individuals recorded from one house in Honduras [7,8], and was consistently associated with much higher rates of *T. cruzi *transmission compared to other Central American vectors such as *T. dimidiata *[8,9].

### *Distribution patterns of *R. prolixus *in Central America and Mexico*

The distribution of *R. prolixus *in Central America followed four main phases corresponding to its initial arrival and spread (1915-1953), early research and initial control trials (1954-1979), further research with larger-scale control attempts (1979-1996), and launch of the IPCA initiative leading to its apparent elimination from the region (1997-2010) (Figure 1).

#### 1915-1953: Discovery and spread

There are no known reports of *R. prolixus *in Central America prior to 1915, when the first specimens were encountered in houses in San Salvador, the capital of El Salvador [5], now believed to have resulted from an accidental laboratory escape [5,6].

From San Salvador, *R. prolixus *appears to have spread initially NW and SE, presumably due to human movements along the international Pan American highway [5], and was first reported in Guatemala in 1934 [10]. Further studies in 1943 confirmed three eastern departments of Guatemala to have house infestations with *R. prolixus *(El Progreso, Esquintla, Jalapa) along with ten departments where *T. dimidiata *was found (Alta Verapaz, Baja Verapaz, Chiquimula, El Progreso, Esquintla, Guatemala, Huehuetenango, Jalapa, Santa Rosa, Zacapa) [11]. Mexico followed in discovering the presence of *R. prolixus *in regions connected to Guatemala by the Pan American highway - in Oaxaca in 1938 [12] and Chiapas in 1949 [13]. Also in 1949, *R. prolixus *was first confirmed in Nicaragua [14] and by 1952 had become distributed throughout the western and central parts of the country, including the departments of Estelí, León, Masaya, Carazo, and Rivas [15]. By 1953, it was also recorded from a few houses in Guanacaste, Costa Rica, in the region bordering Nicaragua [16], but was quickly eliminated from there by insecticide spraying carried out by the antimalaria service of Costa Rica [5,16].

The spread of *R. prolixus *NW and SE from San Salvador does seem to have followed the Pacific route associated with the Pan American highway, such that, for example, it has never reached the Atlantic departments of Nicaragua (RAAN: Región Autónoma del Atlántico Norte, and RAAS: Región Autónoma del Atlántico Sur), nor the Yucatan peninsula or Belize. It arrived in Honduras only during the late 1950s, possibly from neighbouring regions of Guatemala into the western departments of Copán and Santa Barbara, or from El Salvador along the Pan American highway into the southernmost department of Choluteca [17] rather than directly across the mountainous border between El Salvador and Honduras.

#### 1954-1979: Early research and initial control trials

From the 1950s, having recognised the presence of *R. prolixus *as a public health problem, the Central American countries began further investigation on its distribution and susceptibility to insecticides [18,19], including field trials of possible control by indoor residual spraying (IRS) as carried out by the national malaria eradication services [16,20].

In El Salvador, a nationwide control campaign against *R. prolixus *began in 1955 and continued until 1976 [20-23]. Through a series of surveys and IRS interventions, a total of 14 departments (Ahuachapán, Cabañas, Chalatenango, Cuscatlán, La Paz, La Unión, Libertad, Morazán, San Miguel, San Salvador, San Vicente, Santa Ana, Sonsonate, and Usulután) were found to be infested and all were sprayed accordingly [19-22]. In 1956, a baseline entomological survey of 23 villages of 17 municipalities in nine departments identified 14 villages with *R. prolixus *and 12 villages with *T. dimidiata*, with 326 and 244 specimens collected, respectively [20]. In a following study of 25 villages of ten municipalities in six departments during 1973-1975, 17 villages were recorded as infested with *R. prolixus *and/or *T. dimidiata*, noting the percentage of infested houses at 10% for *R. prolixus *and 30.3% for *T. dimidiata *[22]. The same study also recorded the number of collected specimens for *R. prolixus *and *T. dimidiata *as 239 and 437, respectively. Throughout El Salvador from the 1950s to 1970s, *R. prolixus *was observed principally in houses lower than 330 MASL [21,22].

Guatemala also continued with entomological investigations during the 1950s, and by 1959 the presence of *R. prolixus *had been confirmed in the departments of Guatemala, Zacapa, Jutiapa, El Progreso, and San Marcos [24]. In Jutiapa which shares the border with El Salvador, the number of captured specimens of *R. prolixus *was 218, whereas that of *T. dimidiata *was just 40. Zacapa, another eastern department bordering Honduras, showed a similar tendency with 309 *R. prolixus *captured, compared to just 31 *T. dimidiata*. As in El Salvador, *R. prolixus *also seemed to predominate in houses at lower altitudes, although some were found in houses up to 1,200 MASL [24].

In Honduras, the presence of *R. prolixus *was recorded for the first time in 1960, in the departments of Santa Barbara and Francisco Morazán [17]. However, in an entomological survey conducted over 76 villages of 12 departments during 1970-72, 40 villages in nine departments (Intibucá, Copán, La Paz, Santa Barbara, Lempira, Choluteca, Olancho, Francisco Morazán, El Paraíso) were found infested with *R. prolixus*, including 14 villages with both *R. prolixus *and *T. dimidiata *(including the capital city, Tegucigalpa, where a single adult *R. prolixus *was found in a bus terminal, possibly illustrating its main mode of dispersal) [25]. The altitude of the 40 infested villages was between 460 and 1,500 MASL. This survey illustrated the rapid proliferation of *R. prolixus *in Honduras, showing a sudden rise in rural house infestations, often associated with an unexpectedly high frequency of acute Chagas infections. In 1971 for example, in a single house in Francisco Morazán, three brothers simultaneously presented with acute infections (with Romañas sign) and all eight family members proved to be serologically positive for *T. cruzi*; at that time over 600 *R. prolixus *were collected from their house, with similar numbers from neighbouring houses where 50% of the inhabitants showed positive serology for Chagas disease [26]. In 1989 in this same locality (Pueblo Nuevo, Municipality of Cedros) one house was completely dismantled to give a total collection of 11,246 specimens of *R. prolixus *[7,8].

In Mexico, the National Malaria Eradication Campaign began in 1956, based on residual insecticide spraying focused particularly on low-lying villages of the southernmost states [27]. These interventions included the states of Oaxaca and Chiapas, and appear to have also impacted on Chagas disease vectors [28,29]. Surveys during the 1960s and 1970s found *R. prolixus *in only four villages in Oaxaca [30-32] and two villages in Chiapas [33], and in some cases these reports were based on the finding of just a single specimen [30,32] indicating very low vector density.

#### 1980-1996: Continued investigation

Civil wars in Guatemala (1960-1996), Nicaragua (1979-1990), and El Salvador (1980-1992) disrupted much of the entomological research and surveillance. In Honduras however, a nationwide sampling survey was carried out during 1983-1984 [27]. This showed nine of the 14 departments to be infested with *R. prolixus *(Comayagua, Copán, Choluteca, El Paraíso, Francisco Morazán, Lempira, Olancho, Yoro, Santa Barbara) in which 17 of 27 municipalities were infested with *R. prolixus*, with almost all also showing some infestation with *T. dimidiata*. The survey also reported that neither vector was present along the Caribbean coastal areas [34].

The Honduras national survey of 1983-1984 revealed particularly high rates of house infestation and seroprevalence of *T. cruzi *in parts of the department of Choluteca. This region was then chosen by the Ministry of Health for a control trial during 1991-94, focusing on the municipalities of San Marcos de Colón and Duyure. The trial began with a baseline survey of 4,411 houses in the 288 localities of these two municipalities, of which Triatominae were found in 1,103 houses, including 634 infested with *R. prolixus*; an indoor residual spray campaign followed, covering 4,331 houses [35]. Serological studies on 3,229 children less than 5 years old showed 62 seropositives (1.9%) who were treated with nifurtimox; serological examination 20 months later showed that 75.8% of these had become seronegative (C. Ponce, personal communication).

Guatemala restarted investigations in the early 1990s as part of a tropical disease research project with the Japan International Cooperation Agency (JICA). A national entomological survey during 1995-1997 reported *R. prolixus *in five departments (El Progreso, El Quiché, Zacapa, Chiquimula, and Jalapa), of which four are located in the east in accordance with the historical data of the 1950s [36]. Unlike previous surveys however, *R. prolixus *was now only found in villages at altitudes above 600 MASL [36].

El Salvador had suspended all vector control activities during the 1980s, but resumed after the ceasefire in 1992. In 1995, an extensive study over 14 previously endemic departments found no presence of *R. prolixus *[37,38].

In Mexico, studies on vector distribution in endemic areas, as well as nationwide surveillance, continued during the 1980s and 1990s, but *R. prolixus *was rarely reported [e.g. [32,39,40]]. A detailed survey of Triatominae in the state of Oaxaca reported three specimens of *R. prolixus *collected in 1998 from two villages near a previously reported collection site at San José de las Flores [41]; this appears to be the last published record this species in Mexico, although a further specimen was collected in Oaxaca in 2002 (C. Ponce, personal communication, see also [42]).

#### 1997-2010: Regional initiative and apparent elimination

Following technical planning discussions organised by the ECLAT network (European Community Latin America Triatominae research network) [43], the Central American Chagas disease control initiative, IPCA, was launched in 1997 by resolution of the Ministers of Health of Belize, Costa Rica, El Salvador, Guatemala, Honduras, Nicaragua and Panama at their 7^th ^RESSCA (Reunión del Sector Salud de Centroamérica) meeting in Belize [38]. Having recognised Chagas disease as of major public health significance, but feasible to control according to the experiences of South American countries [e.g. [44,45]], the seven countries of Central America established the objective of halting vectorial and transfusional transmission of Chagas disease, including elimination of *R. prolixus *as one of the specific means to achieve this [38]. To initiate the programme, financial aid of 500,000 US dollars was provided by the Government of Taiwan to each Central American country, although much of this was then used for emergency relief following hurricane Mitch in 1998, or for other activities, except in Nicaragua and Honduras.

Under the IPCA initiative, the essential strategy involved revisiting all localities thought to be infested from previous studies, or suspected of possible infestation due to proximity to previously-reported foci or with housing conditions considered at risk to infestation (e.g. houses with palm or thatch roofs). Houses in these localities were checked for infestation by staff of the ETV programmes (Enfermedades Transmitidas por Vectores/Vector-borne Diseases) and/or trained community volunteers, by consultation with the householders and a physical search of the premises for the presence of Triatominae. On initial inspection, the finding of a single live *R. prolixus *in any house was sufficient to declare the whole locality infested, and organise indoor residual spraying (IRS) of all houses and peridomestic habitats in the locality, usually using a 3^rd ^generation pyrethroid such as deltamethrin SC at 25 mg a.i./m^2^, or lambda-cyhalothrin WP at 30 mg a.i./m^2^, following WHO guidelines [46-48]. Subsequently, if a house were again found to be infested through follow-up inspections or community-based surveillance, all houses in the village were resprayed.

Nicaragua was the first country to implement the programme, conducting entomological surveys in 1998-1999, IRS campaigns in 1999-2002, and follow-up surveillance with focal spraying during 2002-2009 [49]. The initial surveys covered 32,195 houses in 129 municipalities of all departments (except RAAN and RAAS where *R. prolixus *had never been reported), revealing the presence of *R. prolixus *in 59 villages of 14 municipalities in eight departments (Carazo, Chinandega, Granada, Jinotega, Madriz, Masaya, Matagalpa, and Nueva Segovia) [49]. The 59 infested villages were at altitudes between 60 and 1,414 MASL, with 8 of these (13.6%) below 600 MASL [49]. By 2002, it appeared that all these infestations had been eliminated by an IRS campaign, but extended surveys during 2002-2004 revealed three further infested localities in the departments of Madriz, Nueva Segovia, and Matagalpa [50]. These newly-discovered infested villages were sprayed during 2007-2008, but again, the subsequent entomological surveillance showed *R. prolixus *in four more villages in Madriz and Nueva Segovia [51]. In 2009, *R. prolixus *was found in one village in Madriz, and this seems to be the last report of this species in Nicaragua [49].

In 1998, Honduras also initiated vector control activities in a few endemic areas of the departments of Santa Barbara and Francisco Morazán. Discovery of villages infested with *R. prolixus *augmented during 1999-2002, when MSF (Médecins sans Frontières) implemented Chagas disease control projects in the departments of Yoro and Francisco Morazán, finding *R. prolixus *in 116 villages in Yoro and 60 villages in Francisco Morazán [52,53]. Alongside the MSF projects, the Honduran Ministry of Health continued finding foci of *R. prolixus *in the departments of Olancho (30 villages), El Paraíso (12 villages), La Paz (5 villages), Choluteca (2 villages), Intibucá (1 village) and Copán (1 village), all of which share borders with Nicaragua, El Salvador or Guatemala.

El Salvador continued with an entomological survey over 162 villages of 14 departments during 1999-2000 [7] and implemented a vector control project with JICA, directed primarily against *T. dimidiata*, that covered seven departments (Ahuachapán, Libertad, Morazán, San Miguel, Santa Ana, Sonsonate, and Usulután) during 2003-2011. A further entomological study covering all 43 municipalities bordering Guatemala or Honduras, searched for possible infestation by *R. prolixus*, but no specimens of this vector were found throughout these surveys [23].

Guatemala carried out vector control projects with JICA through the periods of 2000-2007 and 2009-2012. During these, a total of 317 villages of nine departments reported the presence of *R. prolixus*. The number of infested villages was 230 in Chiquimula, 35 in Zacapa, 29 in Jalapa, 10 in Jutiapa, 8 in El Progreso, 2 in Huehuetenango, 1 in Santa Rosa, 1 in Baja Verapaz, and 1 in El Quiché. Of the 317 villages, 313 (98.7%) were located in the eastern region of the country (mainly bordering Honduras).

During 2003 to 2011, Honduras also intensified vector control interventions with international aid from JICA, CIDA (Canadian International Development Agency), World Vision, and CARE International [54,55]. The number of villages with *R. prolixus *registered during this period reached 70 in Intibucá, 47 in La Paz, 30 in Olancho, 27 in Lempira, 24 in Copán, 19 in Yoro, 8 in Francisco Morazán, 7 in Santa Barbara, 6 in Ocotepeque, 6 in Comayagua, and 4 in El Paraíso. Among the total of 228 villages found infested during 2003-2011, 20 appeared to have been reinfested since control attempts in 2003.

Following the intensified IRS and surveillance campaigns, in 2008 Guatemala became the first country to be certified by IPCA as having interrupted transmission of Chagas disease due to *R. prolixus*. And having evidenced significant reduction in vector distribution and in seroprevalence in children in endemic areas, Nicaragua and Honduras were similarly certified in 2011. Also, because of the apparent absence of *R. prolixus *following repeated surveys and continual surveillance, certification of elimination of this vector was awarded to Mexico in 2009, El Salvador in 2010 and Costa Rica in 2011. In 2010, Honduras was the only country in Central America to report the presence of *R. prolixus *with four infested villages (Figure 1e) and by June of that year these four localities had been resprayed [55]. Since then, there have been no further reports of *R. prolixus *in Central America, and it may be that regional elimination has been achieved (Table 1).

**Table 1:** The rise and fall of *Rhodnius prolixus *in Central America and Mexico.

	First reported presence	Last reported presence	PAHO-IPCA certification	
			*	**
El Salvador	1915	1976		2010
Guatemala	1934	2008	2008	(1)
Mexico	1938	2002		2009
Nicaragua	1949	2009	2011	(2)
Costa Rica	1952	1953		2011
Honduras	1960	2010	2011	(2)
Belize	never encountered			
Panama	never encountered			

* formal certification of interruption of transmission of *Trypanosoma cruzi *due to *Rhodnius prolixus* ** formal certification of elimination of *Rhodnius prolixus*

(1)Guatemala has now completed three years with no reports nor encounters of *R. prolixus *in surveys, and is being considered for formal certification of elimination of this species. (2)Nicaragua and Honduras have now completed almost two years with no reports nor encounters of *R. prolixus *in surveys, and may be considered for certification of elimination if this continues.

## Discussion

The discontinuous distribution of *R. prolixus *between the llanos of Venezuela and Colombia, and various parts of Central America, has long invited speculation. The two forms are genetically similar [6] and in both areas appear to have been of domestic and peridomestic habit, associated particularly, but not exclusively, with houses of palm-thatch roofs. The absence of *R. prolixus *from NW Colombia, Panama, and southern and central Costa Rica, suggests that active migration between the South and Central American populations was not possible, and that passive transport, for example amongst the belongings of travellers, was limited by the difficult access through the Darian region of the isthmus. Gamboa [56,57], on first finding *Rhodnius *populations in palmtree crowns in Venezuela, suggested that the discontinuity might be explained by passive transport of eggs and nymphs by birds (*Mycteria americana*) migrating between Venezuela and Central America but, although often repeated, there is no further evidence to support this idea. The Venezuelan palmtree populations of *Rhodnius *were almost certainly the morphologically similar *R. robustus *(from which the domestic *R. prolixus *may have derived [1]) and, in spite of numerous studies, no populations of *Rhodnius *have been found in Central American palmtree crowns (except for the distinctive *R. pallescens *in Panama, Costa Rica, and parts of Nicaragua).

If the Central American forms of *R. prolixus *originated from an accidental escape from a laboratory-reared colony at the beginning of the last century, then these forms have shown a remarkable capacity for dispersal. Within half a century they reached five countries [2] and we may suppose that, were it not for interventions by the antimalaria services of Costa Rica and Mexico, they may have spread even further. They showed no apparent capacity for colonising silvatic habitats, but readily colonised rural houses and peridomestic habitats, especially those with roofs of palm or thatch, in some cases reaching very high density populations. But unlike the spread of *Triatoma infestans *in Central Brazil during the same period, which seems to have displaced previous domestic infestations of *Panstrongylus megistus *[44,45], there is no evidence that Central American *R. prolixus *were displacing previous infestations of autochthonous Triatominae, and this may have contributed to their apparent ease of domestic dispersal. They appear to have followed the main routes of human migration along the Pacific side of Central America, presumably carried amongst the belongings of travellers and migrant workers. So up to the 1970s, they were mainly found in the more densely populated lowland areas (below 600 MASL) and, since the 1950s, these lowland regions became the main focus for antimalaria interventions by indoor residual spraying (IRS) that appear to have had a substantial impact.

The apparent success of the vector control interventions against *R. prolixus *in Central America thus seems to have been influenced by both biological and operational factors. The history of its accidental release into the region [5], largely confirmed by genetic comparisons [6], suggests that the Central American populations had experienced a series of founder effects and genetic bottlenecks - the original sample collected in Venezuela and reared in Paris, the subsample then taken to El Salvador, and successive subsamples assumed to have been accidentally carried to other countries in association with human migrations, leading to a genetically restricted form showing relatively low variability [6] and hence low likelihood of selection for new attributes such as insecticide resistance. These Central American populations then showed high susceptibility to insecticides, particularly to pyrethroids [58,59], and it seems likely that antimalarial IRS campaigns launched during the 1950s-60s would have contributed significantly to their control. Even the widespread use of DDT during the antimalaria campaigns could have contributed, because although DDT is generally considered ineffective against *Triatoma *[60] it has been shown to have at least a latent effect against *R. prolixus *in Venezuela [61], and seems likely to have had a more significant effect on the smaller and genetically-restricted Central American forms of this species. This idea is reinforced by the initial disappearance of *R. prolixus *from the lower altitude villages, where antimalaria campaigns were most intense, and also by its apparent disappearance from Mexico where there have been few control campaigns specifically directed against Triatominae.

In Central America, despite numerous studies, *R. prolixus *was never encountered in silvatic habitats, and appears to have been confined to domestic and peridomestic habitats, especially houses with roofs of palm or thatch. In addition to the insecticide spraying, it seems likely that house improvement may have contributed to reducing the vector distribution. Especially over the last two decades, thatched roofs have tended to be replaced with roofs of tile or corrugated metal - although *R. prolixus *has been found in the walls of tin roofed houses in Honduras and Guatemala [54], and it has been demonstrated that house improvement alone is generally insufficient to eliminate domestic populations of Triatominae [e.g. [62]].

But the key factors in the successful control of *R. prolixus *in Central America have been the technical recognition of its importance as a public health problem and the feasibility of its successful control [43] followed by commitment of the National Governments, together with technical and financial support from other agencies. Initial commitment by researchers led to the discovery of *R. prolixus *and documentation of the potential magnitude of Chagas disease as a public health issue. This alert was responded to by the Salvadoran and Costa Rican governments by investing in vector control operations that had a substantial impact during the 1950s-1970s. From the 1980s to early 1990s however, the government commitment to Chagas disease control was negligible in Guatemala, Nicaragua and El Salvador, mainly due to the political unrest. The Ministry of Health of Honduras continued with serological and entomological studies, mostly with external funds, and provided important data that helped to vitalise interest in a regional Chagas disease control programme. In the early 1990s, the Guatemalan national university restarted entomological survey work with financial and technical assistance from JICA, such that these two centres; the MoH of Honduras, and the Universities of Guatemala, became the main technical centres supporting the regional control efforts (See acknowledgements).

Establishment of IPCA in 1997 was a turning point, backed by scientific consensus [43], political commitment with coordination from the Pan American Health Organization [38], and supported by international organizations (JICA, CIDA) and NGOs (MSF, CARE, PLAN and World Vision). As pledged by the member countries, the focus of commitment was altered from investigation to operational interventions designed to achieve the specific objectives, including elimination of *R. prolixus*. The clear objectives of the IPCA programme facilitated the mobilisation of additional national resources, and also promoted participation of international donors with operational, managerial, technical and financial assistance. Involvement of the external stakeholders further contributed to raise the priority level of Chagas disease control within the Ministries of Health, improving resource allocation and gradually integrating the activities into existing programmes of epidemiological surveillance and vector control. Through support from the ECLAT network and PAHO, much was learned from the experience of other countries, especially in relation to the control of *Triatoma infestans *in the southern cone countries [e.g. [43-45]], and the IPCA annual meetings and thematic workshops also paved the way for definition of criteria for certification of interruption and elimination of Chagas disease transmission [e.g. [7]]. To a certain extent, the IPCA workshops also encouraged competition in achieving the objectives, through open discussion of the annual achievements of each of the national programmes.

Although it now appears that by the mid-1990s the overall distribution of *R. prolixus *in Central America had been reduced by antimalaria IRS interventions, especially in El Salvador, Costa Rica, and Mexico, and may also have been declining in some areas through local house improvement, it remained the most significant vector of Chagas disease throughout the region [7-9]. In 1990, it was estimated from serological surveys that over 1.77 million people were infected with *T. cruzi *in Central America, implying an overall incidence of nearly 62,000 new cases per year [63]. But by 2006, following the main vector control campaigns, estimates by the Pan American Health Organization suggested that infection prevalence in the region had declined to 806,000, with just 8,500 new cases per year attributable to vector-borne transmission (mainly due to residual infestations with *T. dimidiata*) [64,65]. With the apparent elimination of *R. prolixus*, these estimates can be expected to decline further.

## Conclusion

*R. prolixus *appears to have been accidentally released into Central America early in the last century, and spread rapidly to become the most serious vector of Chagas disease in the mesoamerican region. Since the 1950s, indoor residual spray campaigns by the malaria eradication services appear to have halted its spread, and reduced its distribution in lowland areas, but most interventions were suspended during the civil unrest of the 1980s. In 1997, the Central American countries launched a multinational initiative against Chagas disease (IPCA) that included elimination of *R. prolixus *amongst the main objectives. The clear objectives and strong political mandate attracted external support (especially from the Japanese Cooperation, JICA) and facilitated operational planning and implementation of large-scale vector surveillance and control interventions. By August 2011 all the previously endemic countries of Central America had been certified as free of Chagas disease transmission due to *R. prolixus*, and it may be that this vector has now been eliminated from the mesoamerican region.

## List of Abbreviation

**CIDA: **Canadian International Development Agency; **ECLAT: **European Community Latin America Triatominae research network; **ETV: **Enfermedades Transmitidas por Vectores; **IPCA: **Iniciativa de los Países de Centro América para la Interrupción de la Transmisión Vectorial, Transfusional y Atención Médica de la Enfermedad de Chagas; **IRS: **Indoor residual spraying; **JICA: **Japan International Cooperation Agency; **MASL: **Meters above Sea Level; **MSF: **Médecins sans Frontières; **PAHO: **Pan American Health Organization; **RAAN: **Region Autónoma del Atlántico Norte (North Atlantic Autonomous Region of Nicaragua); **RAAS: **Región Autónoma del Atlántico Sur (South Atlantic Autonomous Region of Nicaragua); **RAPD: **Random Amplification of Polymorphic DNA; **RESSCA: **Reunión del Sector Salud de Centroamérica.

## Competing interests

The authors declare that they have no competing interests.

## Authors' contributions

KH and CJS conceived the review design, wrote the drafts and approved the final manuscript.
